# Risk Factors for Non-Carbapenemase-Producing Carbapenem-Resistant *Enterobacterales* Infections: A Retrospective Cohort Study

**DOI:** 10.3390/geriatrics10030069

**Published:** 2025-05-22

**Authors:** Sayaka Mabuchi, Tsukasa Nakamura, Toshihiro Imada, Junji Mashino, Takeshi Morimoto

**Affiliations:** 1 Department of Human Resources Development for General Practitioner, Shimane Prefectural Central Hospital, Izumo 693-0068, Japan; m14121sy@jichi.ac.jp; 2 Department of Infectious Diseases, Shimane Prefectural Central Hospital, Izumo 693-0068, Japan; tsukanam@spch.izumo.shimane.jp; 3 Department of General Medicine, Shimane Prefectural Central Hospital, Izumo 693-0068, Japan; t-imada@spch.izumo.shimane.jp (T.I.); mashino42@spch.izumo.shimane.jp (J.M.); 4 Department of Clinical Epidemiology, Hyogo College of Medicine, Nishinomiya 663-8501, Japan

**Keywords:** carbapenem resistant, *Enterobacterales*, carbapenemase-producing *Enterobacterales*, antibiotics

## Abstract

**Background/Objectives:** Carbapenem-resistant Enterobacterales (CRE) infections are widespread, and the risk factors for carbapenemase-producing CRE (CP-CRE) infections are known. Non-CP-CRE (NCP-CRE) infections occur frequently; however, the associated risk factors remain elusive. Therefore, we investigated the risk factors for NCP-CRE infections, especially those caused by Enterobacter and Citrobacter species. **Methods:** We conducted a retrospective cohort study of patients aged ≥ 18 years with Enterobacter or Citrobacter infections who were admitted to the Department of General Medicine of a tertiary care hospital in Japan from October 2014 to September 2020. We used the data at first detection and performed univariate and multivariate logistic regression analyses to assess the associations between NCP-CRE infections and risk factors such as patient characteristics and antibiotics. **Results:** In total, 1416 participants were evaluated. The mean age of the patients was 74 ± 17 (range: 18–107) years, of whom 746 (53%) were men. Past use of antibiotics (≥4 days before specimen collection) was not significantly associated with NCP-CRE infections (133 [84%] vs. 1034 [82%], *p* = 0.5); however, recent use (≤3 days before sample collection) was significantly associated with NCP-CRE infections (42 [27%] vs. 245 [19%], *p* = 0.036). In the multivariate logistic model, recent use of antibiotics (odds ratio: 1.50, 95% confidence interval: 1.03–2.18) was an independent risk factor for NCP-CRE infections. **Conclusions:** NCP-CRE infection may be associated with recent antibiotic exposure, but not with the host’s immune status. Therefore, alternative risk factors for NCP-CRE infection may exist.

## 1. Introduction

Carbapenem-resistant *Enterobacterales* (CRE) infections are widespread and pose a serious health problem globally [1]. CRE can be divided into two categories: carbapenemase-producing CRE (CP-CRE) and non-carbapenemase-producing CRE (NCP-CRE). Carbapenem resistance occurs because of the production of carbapenemase or a combination of β-lactamase [especially extended-spectrum β-lactamase (ESBL) and AmpC] and structural mutations [2,3]. CP-CRE infections spread at a faster rate because carbapenemase is plasmid-mediated. The two main risk factors for CP-CRE infections are antibiotic exposure (e.g., to carbapenems and cephalosporins) and host immune status [4,5,6].

Although NCP-CRE infections occur frequently worldwide, only a few studies have reported their occurrence. NCP-CRE infections are often detected at our hospital; however, there have been no cases of CP-CRE infections since the start of this study in 2014. Although the incidence of NCP-CRE infection is higher than that of CP-CRE infection, the risk factors for NCP-CRE infection remain poorly understood, as most published studies have focused on CRE or carbapenemase-producing *Enterobacterales* (CPE) [7]. Generally, CPE includes *Klebsiella* [8,9], *Enterobacter*, *Citrobacter* species, and *Escherichia coli*.

Several studies on CRE infections have been conducted in hospitals and intensive care units (ICUs), but it is unclear whether a nosocomial source is a risk factor for NCP-CRE infections. We investigated the risk factors for NCP-CRE infections by comparing them to infections with carbapenem-susceptible *Enterobacterales* (CSE). We specifically examined *Enterobacter* and *Citrobacter* species, which are opportunistic infectious bacteria that are often detected in our hospital.

## 2. Materials and Methods

### 2.1. Study Design and Patients

We conducted a retrospective cohort study of adult patients (aged ≥ 18 years) who were identified as having *Enterobacter* or *Citrobacter* infections between 1 October 2014, and 30 September 2020, at Shimane Prefectural Central Hospital, a tertiary care facility in Japan. The specimens included sputum, urine, blood, pharyngeal and laryngeal samples, and wound exudate. The inclusion criterion was a positive culture result for *Enterobacter* or *Citrobacter*. We collected data from both inpatients and outpatients, beginning at the time of the first detection of Enterobacter or Citrobacter species. There were no exclusion criteria for this study.

We collected the study data from the Shimane Prefectural Central Hospital Integrated Intelligent Management System database. This study was approved by the Institutional Review Board of Shimane Prefectural Central Hospital. As all data were obtained as part of our routine daily practice, the need for informed consent was substituted by an opt-out method, as approved by the institutional review board.

### 2.2. Measurements

Clinical data included age, sex, body mass index (BMI), patient information (smoking history, history of hospitalization, history of ICU stay, history of surgical operation, past solid organ transplantation, and previous radiation therapies), hospitalization status (hospital-acquired infections, ICU stay, pregnancy, endoscopy, central vein catheter, mechanical ventilation, and Foley catheter), comorbidities (malignancy, diabetes, connective tissue diseases, chronic obstructive pulmonary disease [COPD], cardiac disease, trauma, and dialysis), and medications (chemotherapy, corticosteroids, and immunosuppressants). Detection of specimens after 3 days of hospitalization was defined as nosocomial infection. We also collected data on patient vital signs (systolic blood pressure [SBP], diastolic blood pressure [DBP], heart rate, respiratory rate, and body temperature) and laboratory test results (white blood cell count [WBC], hemoglobin, platelet cell count [Plt], serum albumin [Alb], aspartic aminotransferase [AST], alanine aminotransferase [ALT], blood urea nitrogen [BUN], creatinine [Cr], estimated glomerular filtration rate [eGFR], sodium, potassium, and C-reactive protein [CRP]) at the time of culture inspection. The date of vital sign and laboratory test measurement was the date of specimen collection.

We also collected specific types of cultures and detection numbers according to the sample source and bacterial resistance (AmpC, ESBL, meropenem-resistant, imipenem-resistant, and cefmetazole-resistant). We collected data on antibiotic use, including past and recent antibiotic use (≥4 days and ≤3 days before specimen collection, respectively), as well as the types of antibiotics used. For patients with current antibiotic use, we separated those who were within 3 days of sample collection and assigned them as patients with recent antibiotic use.

### 2.3. Outcome Measures

We defined CRE as “resistance to meropenem” or “resistance to imipenem and cefmetazole”. The minimum inhibitory concentration of meropenem or imipenem was ≥2 μg/mL, whereas that of cefmetazole was ≥64 μg/mL. All isolates were screened for carbapenemase, ESBL, and AmpC production. We used modified carbapenemase inactivation method (mCIM) for carbapenemase and inhibitor-based combination disk test (CDST) for carbapenemase, ESBL, and AmpC. Product names were MASTDISCS COMBI CARBA PLUS and AMPC ESBL DETECTION SET (Mast Group). For patients with recent antibiotic use, specimens were collected 3 days after antibiotic use and assigned to the hospital infection group. If carbapenem-susceptible *Enterobacterales* (CSE) and CRE were detected in one person on the same day, we only used the CRE data.

### 2.4. Statistical Analysis

Continuous variables are presented as the mean and standard deviation (SD) or median and interquartile range (IQR). Categorical variables are presented as numbers and percentages. Continuous variables were compared using Student’s *t*-test or Wilcoxon’s rank-sum test based on distribution. Categorical variables were compared using the χ^2^ test or Fisher’s exact test. The variables evaluated in the multivariate logistic model were risk factors for CP-CRE infection in the univariate analysis and included recent antibiotic use, past antibiotic use, heart disease, smoking, male sex, chemotherapy, corticosteroids, or proton pump inhibitors (PPIs). After constructing the model with all variables, backward selection was conducted to ensure that all variables were significantly associated for exploratory analyses. All statistical analyses were performed using JMP 12.2.0 and Stata 14 (StataCorp LLC, College Station, TX, USA). Two-tailed *p*-values < 0.05 were considered indicative of statistical significance.

## 3. Results

### 3.1. Patient Characteristics

In total, 1416 patients were evaluated (Table 1). The mean age of the patients was 74 (SD: 17) years (range: 18–107 years), of whom 746 (53%) were male. The patient’s BMI was 21 ± 3.7 kg/m^2^ (range: 18–23 kg/m^2^). Furthermore, 605 (44%) patients had a smoking history and 997 (70%) a history of hospitalization, including 254 (18%) with a history of ICU stay. In total, 625 (44%) patients had hospital-acquired infections, 796 (56%) had a surgical history, and 107 (7.6%) were pregnant. Additionally, 83 (5.9%) patients had a history of radiation therapy, 652 (46%) had a history of endoscopy, and 149 (11%) died during their hospital stay. Regarding comorbidities, 445 patients (22%) had cancer, 566 (40%) diabetes mellitus, 74 (5.2%) connective tissue disease, 116 (8.2%) COPD, 560 (40%) heart disease, 835 (59%) trauma, and 46 (3.2%) were on dialysis. Additionally, 222 (14%) patients had a history of anticancer drug use, 225 (16%) a history of steroid use for at least 14 consecutive days, 35 (2.5%) a history of immunosuppressant use, and 675 (48%) a history of using PPIs. In terms of invasive procedures, five patients (0.35%) used central vein catheters, 197 patients (14%) mechanical ventilators, and 161 patients (11%) Foley catheters.

The mean SBP was 124 mmHg (SD: 26 mmHg), the mean DBP was 71 mmHg (SD: 17 mmHg), the mean heart rate was 88 beats per min (SD: 20 beats per min), the mean respiratory rate was 22 breaths per min (SD: 6.5 breaths per min), the mean body temperature was 37.2 °C (SD: 1.1 °C), and the median hemoglobin concentration was 11.3 (IQR 9.7–12.7) g/dL.

### 3.2. Organisms

*Enterobacter* species were detected in 945 (67%) participants, and *Citrobacter* species were detected in 471 (33%) participants (Table 2). The most common specimen collected was sputum (487 [34%]), followed by urine (422 [30%]), blood (87 [6.1%]), pharyngeal/laryngeal secretion (65 [4.6%]), wound (82 [5.8%]), and others (273 [19%]). Two patients (0.14%) had meropenem-resistant isolates, 184 (13%) had imipenem-resistant isolates, and 904 (64%) had cefmetazole-resistant isolates. Carbapenemase production was not detected in any of the samples.

### 3.3. Antibiotic Use

A total of 1167 (82%) patients received antibiotic treatment in this study, 287 (20%) of whom had recently used antibiotics (Table 3). Additionally, 121 (8.5%) patients had recently used first- or second-generation cephalosporins, 14 (1.0%) had recently used oral third-generation cephalosporins, and 21 (1.5%) had recently used intravenous third-generation cephalosporins. None of the patients had recently used fourth-generation cephalosporins.

### 3.4. Univariate Association with NCP-CRE

A total of 158 (11%) patients had an NCP-CRE infection. The proportion of males in NCP-CRE was higher than that of CSE (97 [61%] vs. 649 [52%], *p* = 0.02) (Table 4). Patients with NCP-CRE infection were older than those with CSE infection (76 ± 15 vs. 74 ± 18 years) without reaching statistical significance (*p* = 0.2). Patients with a smoking history were more likely to have NCP-CRE infections (78 [53%] vs. 527 [43%], *p* = 0.03). There was also a statistically significant relationship between hospital-acquired infections and NCP-CRE infection (80 [51%] vs. 545 [43%], *p* = 0.0081). Regarding invasive procedures, there were no significant differences (mechanical ventilators: 25 [16%] vs. 172 [14%], *p* = 0.5; Foley catheters: 14 [8.9%] vs. 147 [12%], *p* = 0.3). The median hemoglobin levels were significantly lower in patients with NCP-CRE infection than in those with CSE infection (median: 10.8 g/dL; IQR: 9.4–12.3, minimum: 5.1, and maximum: 16.4 vs. median: 11.3 g/dL; IQR: 9.7–12.8, minimum: 4.9, and maximum: 22.7, *p* = 0.032).

There were no significant differences in the previous use of antibiotics between NCP-CRE and CSE (158 [11%] vs. 1258 [89%], *p* = 0.5). Recent antibiotic use was significantly more common in patients with NCP-CRE infection than in those with CSE infection (42 [27%] vs. 245 [19%], *p* = 0.036) (Table 4). Recent use of first- or second-generation cephalosporins was more common in patients with NCP-CRE than in those with CSE (22 [14%] vs. 99 [7.9%], *p* = 0.01). There were significant differences in the recent use of third-generation oral cephalosporins (five patients with NCP-CRE infection [3.2%] and nine with CSE infection [0.72%], *p* = 0.014). There were no significant differences in the recent use of carbapenems (5 [3.2%] vs. 18 [1.4%], *p* = 0.2).

### 3.5. Multivariate Association with NCP-CRE

The multivariate logistic model showed that recent antibiotic use [odds ratio (OR): 1.29, 95% confidence interval (CI): 0.86–1.94] and history of antibiotic use (OR: 1.23, 95% CI: 0.74–2.05) were not relevant to NCP-CRE infections (Table 5). Furthermore, heart disease (OR: 1.21, 95% CI: 0.84–1.73), chemotherapy (OR: 0.87, 95% CI: 0.55–1.38), corticosteroid use (OR: 1.06, 95% CI: 0.66–1.70), PPI use (OR: 0.92, 95% CI: 0.63–1.32), smoking (OR: 1.20, 95% CI: 0.77–1.87), and male sex (OR: 0.94, 95% CI: 0.66–1.34) were not significant risk factors. The backward selection model revealed that only recent antibiotic use (OR: 1.50, 95% CI: 1.03–2.18) was significant.

## 4. Discussion

NCP-CRE infection may be associated with the recent use of antibiotics; however, it is not strongly associated with host immune status. In this study, we showed that 158 (11%) patients had NCP-CRE infection, with no carbapenemase detected in our hospital. In the univariate analysis, recent antibiotic use was a risk factor for NCP-CRE infection. However, based on the multivariate analysis, NCP-CRE infection was not strongly associated with antibiotic exposure or host immune status.

Antibiotic use was not significantly associated with NCP-CRE infection. Previous studies [2,10] have reported that antibiotic exposure, especially exposure to broad-spectrum cephalosporins and/or carbapenems, is an important risk factor for CRE infection or colonization. In this study, there was a significant difference in the use of first- or second-generation cephalosporins, but not carbapenems. As first- or second-generation cephalosporins were often used in our hospital, selection bias may have existed. Most *Enterobacter* and *Citrobacter* species have chromosomal *ampC* genes and produce AmpC enzymes. They have a gene that suppresses the chromosomal ampC genes at the same time, so the production of AmpC is suppressed to a small amount, and they are susceptible to cephalosporins. However, there are mutant strains in which AmpC is constantly produced at high levels due to the inability of the suppressor gene to function as a result of genetic mutation. Such mutant strains may lose susceptibility during their administration. Even if *Enterobacter* and *Citrobacter* species with AmpC are resistant to cephalosporins, they may exhibit susceptibility to cephalosporins once again after their administration has ceased [11]. Therefore, recent antibiotic use, rather than past use, was a significant risk factor.

Furthermore, our findings suggested that factors related to host immune status, such as comorbidities, medications, and invasive procedures, showed less association as risk factors. We also found little difference between inpatients and outpatients with NCP-CRE infections. Other risk factors included hospitalization, malignant diseases, immunosuppression, corticosteroid use, organ transplantation, mechanical ventilation, and the presence of Foley or central venous catheters. Some studies [12,13,14] compared CP-CRE infections with NCP-CRE infections to identify risk factors for CP-CRE. The authors suggested that prolonged hospitalization was a risk factor that may not be strongly associated with host immune status. Our study revealed that the host immune status was less relevant to NCP-CRE infection, too. Therefore, NCP-CRE infection is not something that only immunocompromised individuals need to be aware of.

This study has some limitations. First, the retrospective design of the study made it difficult to collect complete data, particularly the histories of long-term antibiotic use. We also did not collect certain clinical data (e.g., alcohol use). However, previous studies have not considered these factors [15]. We recognize that our available data were within the margin of error. Therefore, other risk factors might exist, and adjustment for unknown confounding factors is not possible. Second, some doctors prescribed antibiotics before identifying the causative organism and its susceptibility. Third, although *Enterobacter* and *Citrobacter* species reside within the intestine, as in many clinical situations, the intestinal environment was not investigated. Fourth, this study did not determine whether the culture result represents colonization or infection when the positive cultures of urine. To understand the risk of NCP-CRE infection, infection or colonization should be assessed on a case-by-case basis.

In summary, the risk factors for NCP-CRE infections differed from those for CP-CRE infections. NCP-CRE infection may not be associated with antibiotic exposure and host immune status.

## Figures and Tables

**Table 1:** Patient characteristics and outcome measures.

Clinical Characteristics	*n* = 1416
Male sex	746 (53)
Age (years)	74 ± 17
BMI (kg/m^2^)	21 ± 3.7
Smoking history	605 (44)
History of hospitalization	997 (70)
History of ICU stay	254 (18)
Operation	796 (56)
Past solid organ transplantation	134 (9.5)
Hospital-acquired infections	625 (44)
ICU stay	400 (26)
Pregnancy	107 (7.6)
Endoscopy	652 (46)
Previous radiation therapies	83 (5.9)
Comorbidities	
Malignant diseases	445 (31)
Diabetes mellitus	566 (40)
Connective tissue diseases	74 (5.2)
COPD	116 (8.2)
Heart diseases	560 (40)
Trauma	835 (59)
Dialysis	46 (3.2)
Medication	
Chemotherapy	222 (14)
Corticosteroids	225 (16)
Immunosuppressants	35 (2.5)
Proton pump inhibitors	675 (48)
Invasive procedure	
Central vein catheter	5 (0.35)
Mechanical ventilation	197 (14)
Foley catheter	161 (11)
Vital signs	
Systolic blood pressure (mmHg)	124 ± 26
Diastolic blood pressure (mmHg)	71 ± 17
Heart rate (beats/min)	88 ± 20
Respiratory rate (breaths/min)	22 ± 6.5
Body temperature (°C)	37.2 ± 1.1
Laboratory data	
White blood cell count (cells/μL)	9325 (6370–12,718)
Hemoglobin (g/dL)	11.3 (9.7–12.7)
Platelet count (×10^4^/μL)	20 (15–26)
Albumin (g/dL)	3.0 (2.5–3.4)
AST (U/L)	26 (18–45)
ALT (U/L)	18 (11–38)
BUN (mg/dL)	18 (13–26)
Creatinine (mg/dL)	0.8 (0.6–1.0)
eGFR (mL/min/1.73 m^2^)	67 (45–89)
Sodium (mEq/L)	137 (134–140)
Potassium (mEq/L)	4 (3.7–4.4)
CRP (mg/dL)	4.8 (1.7–10)
Outcomes	
In-hospital death	149 (11)

Data are presented as mean ± SD, median (IQR), or *n* (%). BMI: Body mass index, ICU: Intensive care unit, COPD: Chronic obstructive pulmonary disease, AST: Aspartate aminotransferase, ALT: Alanine aminotransferase, BUN: Blood urea nitrogen, eGFR: Estimated glomerular filtration rate, CRP: C-reactive protein, SD: Standard deviation, IQR: Interquartile range.

**Table 2:** Types of microorganisms detected.

	*Enterobacter* Species (*n* = 945)	*Citrobacter* Species (*n* = 471)
	*n* (%)	*n* (%)
Specimens		
Sputum	407 (43)	80 (17)
Urine	210 (22)	212 (45)
Blood	62 (6.6)	25 (5.3)
Pharynx/larynx	56 (5.9)	9 (1.9)
Wound	57 (6.0)	25 (5.3)
Other	153 (16)	120 (25)
AmpC	26 (2.8)	8 (1.7)
ESBL	2 (0.2)	0 (0)
Meropenem-resistant	2 (0.2)	0 (0)
Imipenem-resistant	169 (18)	15 (3.2)
Cefmetazole-resistant	834 (88)	70 (15)

ESBL: Extended-spectrum β-lactamase.

**Table 3:** Antibiotics used.

Antibiotics	*n* = 1416
Past antibiotics	1167 (82)
Penicillin	520 (37)
First- or second-generation cephalosporin	834 (59)
Intravenous third-generation cephalosporin	330 (23)
Oral third-generation cephalosporin	612 (43)
Fourth-generation cephalosporin	87 (6.1)
Monobactam	2 (0.14)
Carbapenem	264 (19)
Active MRSA antibiotics	128 (9.0)
Aminoglycoside	23 (1.6)
Quinolone	194 (14)
Macrolide	38 (2.7)
Tetracycline	78 (5.5)
Metronidazole	67 (4.7)
Trimethoprim/sulfamethoxazole	104 (7.3)
Antituberculosis drugs	19 (1.3)
Recent antibiotics	287 (20)
Penicillin	71 (5.0)
First- or second-generation cephalosporin	121 (8.5)
Intravenous third-generation cephalosporin	21 (1.5)
Oral third-generation cephalosporin	14 (1.0)
Fourth-generation cephalosporin	0 (0)
Monobactam	0 (0)
Carbapenem	23 (1.6)
Active MRSA antibiotics	24 (1.7)
Aminoglycoside	1 (0.07)
Quinolone	0 (0)
Macrolide	28 (2.0)
Tetracycline	4 (0.28)
Metronidazole	8 (0.57)
Trimethoprim/sulfamethoxazole	8 (0.57)
Antituberculosis drugs	5 (0.35)

Data are presented as *n* (%), mean ± SD, or median (IQR). MRSA: Methicillin-resistant *Staphylococcus aureus*, SD: Standard deviation, IQR: Interquartile range.

**Table 4:** Univariate risk factors for NCP-CRE and CSE infection.

	NCP-CRE (*n* = 158)	CSE (*n* = 1258)	*p*-Value
Clinical characteristics			
Male sex	97 (61)	649 (52)	0.020
Age (years)	76 ± 15	74 ± 18	0.16
BMI (kg/m^2^)	21 ± 3.6	21 ± 3.8	0.07
Smoking history	78 (53)	527 (43)	0.030
History of hospitalization	108 (68)	889 (71)	0.55
History of ICU stay	24 (15)	230 (18)	0.34
Past operation	95 (60)	701 (56)	0.57
Hospital-acquired infections	80 (51)	545 (43)	0.08
ICU stay	42 (27)	320 (25)	0.76
Endoscopy	76 (48)	576 (46)	0.58
Comorbidities			
Malignant diseases	57 (36)	388 (31)	0.18
Diabetes mellitus	57 (36)	509 (40)	0.29
Connective tissue diseases	8 (5.1)	66 (5.3)	0.92
Heart diseases	70 (44)	490 (39)	0.19
Trauma	92 (58)	743 (59)	0.84
Medication			
Chemotherapy	26 (16)	196 (16)	0.78
Corticosteroids	26 (16)	199 (16)	0.84
Immunosuppressants	6 (3.8)	29 (2.3)	0.27
Proton pump inhibitors	76 (48)	599 (48)	0.90
Past antibiotics	133 (84)	1034 (82)	0.54
Recent antibiotics	42 (27)	245 (19)	0.036
Invasive procedures			
Mechanical ventilation	25 (16)	172 (14)	0.46
Foley catheter	14 (8.9)	147 (12)	0.29
Vital signs			
Systolic blood pressure (mmHg)	126 ± 25	124 ± 26	0.28
Diastolic blood pressure (mmHg)	70 ± 14	71 ± 17	0.98
Heart rate (beats/min)	87 ± 18	88 ± 20	0.45
Respiratory rate (breaths/min)	21 ± 6.1	22 ± 6.5	0.55
Body temperature (°C)	37.3 ± 1.2	37.3 ± 1.1	0.36
Laboratory data			
White blood cell count (cells/μL)	8390 (5970–11,815)	9410 (6370–12,840)	0.16
Hemoglobin (g/dL)	10.8 (9.4–12.3)	11.3 (9.7–12.8)	0.032
Platelet count (×10^4^/μL)	19 (14–24)	20 (15–26)	0.09
Albumin (g/dL)	3 (2.6–3.4)	3 (2.5–3.4)	0.56
AST (U/L)	25 (18–40)	26 (18–46)	0.39
ALT (U/L)	19 (10–27)	18 (11–39)	0.32
BUN (mg/dL)	17 (12–26)	19 (13–26)	0.22
Creatinine (mg/dL)	0.70 (0.59–0.98)	0.79 (0.59–1.1)	0.20
eGFR (mL/min/1.73 m^2^)	70 (54–89)	66 (45–89)	0.24
Sodium (mEq/L)	137.6 (134.6–140.4)	136.8 (134.2–139.7)	0.13
Potassium (mEq/L)	3.9 (3.7–4.3)	4.0 (3.7–4.5)	0.12
CRP (mg/dL)	5.2 (1.4–11)	4.8 (175–10)	0.72

BMI: Body mass index, ICU: Intensive care unit, AST: Aspartate aminotransferase, ALT: Alanine aminotransferase, BUN: Blood urea nitrogen, eGFR: Estimated glomerular filtration rate, CRP: C-reactive protein, NCP-CRE: Non-carbapenemase-producing carbapenem-resistant *Enterobacterales*, CSE: Carbapenem-susceptible *Enterobacterales*.

**Table 5:** Multivariate analysis of NCP-CRE infection.

	Univariate		Multivariate		Backward	
Variables	OR (95% CI)	*p*-Value	OR (95% CI)	*p*-Value	OR (95% CI)	*p*-Value
Recent antibiotic use	1.50 (1.03–2.18)	0.035	1.29 (0.86–1.94)	0.22	1.50 (1.03–2.18)	0.036
Past antibiotic use	1.33 (0.87–2.02)	0.19	1.23 (0.74–2.05)	0.42		
Heart diseases	1.28 (0.93–1.78)	0.13	1.21 (0.84–1.73)	0.30		
Chemotherapy	1.06 (0.71–1.59)	0.78	0.87 (0.55–1.38)	0.55	1.01 (0.67–1.53)	0.96
Corticosteroids	1.08 (0.70–1.68)	0.72	1.06 (0.66–1.70)	0.83		
Proton pump inhibitors	1.08 (0.78–1.49)	0.64	0.92 (0.63–1.32)	0.64		
Smoking	1.49 (1.06–2.09)	0.020	1.20 (0.77–1.87)	0.42		
Male sex	1.47 (1.06–2.04)	0.021	1.32 (0.84–2.07)	0.23		

OR: Odds ratio, CI: Confidence interval.

## Data Availability

All data relevant to the study are included in the article.

## Abbreviations

The following abbreviations are used in this manuscript:

Alb	Serum albumin
ALT	Alanine aminotransferase
AST	Aspartic aminotransferase
BMI	Body mass index
BUN	Blood urea nitrogen
CP-CRE	Carbapenemase-producing carbapenem-resistant *Enterobacterales*
CPE	Carbapenemase-producing *Enterobacterales*
Cr	Creatinine
CRE	Carbapenem-resistant *Enterobacterales*
CRP	C-reactive protein
CSE	Carbapenem-susceptible *Enterobacterales*
DBP	Diastolic blood pressure
eGFR	Estimated glomerular filtration rate
ICU	Intensive care units IQR interquartile range
NCP-CRE	Non-carbapenemase-producing carbapenem-resistant *Enterobacterales*
CSE	Carbapenem-susceptible *Enterobacterales*
OR	Odds ratio
Plt	Platelet cell count
PPIs	Proton pump inhibitors
SBP	Systolic blood pressure
SD	Standard deviation
WBC	White blood cell count
